# Association of two obesity-related gene polymorphisms LEPG2548A rs7799039 and LEPRQ223R rs1137101 with the risk of breast cancer

**DOI:** 10.18632/oncotarget.19580

**Published:** 2017-07-26

**Authors:** Hui Luan, Hong Zhang, Ying Li, Ping Wang, Lifei Cao, Honglan Ma, Qing Cui, Gang Tian

**Affiliations:** ^1^ Department of Cardiovasology, First Affiliated Hospital, School of Medicine, Xi’an Jiaotong University, Xi’an, Shaanxi, China; ^2^ Department of Oncology, Second Affiliated Hospital, School of Medicine, Xi’an Jiaotong University, Xi’an, Shaanxi, China; ^3^ Department of Neurology, Second Affiliated Hospital, School of Medicine, Xi’an Jiaotong University, Xi’an, Shaanxi, China

**Keywords:** breast cancer, leptin, leptin receptor, polymorphisms

## Abstract

Many studies have been performed to investigate the correlation of leptin (LEP) and leptin receptor (LEPR) polymorphisms with breast cancer (BC) risk, however the results are inconclusive. To obtain a more precise estimation, we conducted this meta-analysis. We searched PubMed, EMBASE, and Web of Science databases to identify qualified studies. Pooled odds ratios (ORs) with 95% confidence intervals (CIs) were used to evaluate the association. Eight eligible studies (2,124 cases and 5,476 controls) for LEP G2548A (rs7799039) polymorphism, and thirteen studies (5,282 cases and 6,140 controls) for LEPR Q223R (rs1137101) polymorphism were included in our study. In general, no significant association between LEP G2548A polymorphism and BC susceptibility was found among five genetic models. In the stratified analysis by ethnicity and sources of controls, significant associations were still not detected in all genetic models. For LEPR Q223R polymorphism, we observed that the association was only statistically significant in Asians (G versus A: OR = 0.532, *P* = 0.009; GG versus AA: OR = 0.233, *P* = 0.002; GA versus AA: OR =0.294, *P* = 0.006; GG versus AA+AG: OR =0.635, *P* = 0; GA+GG versus AA: OR = 0.242, *P* = 0.003), but not in general populations and Caucasians. In conclusion, LEP G2548A polymorphism has no relationship with BC susceptibility, while LEPR Q223R polymorphism could decrease BC risk in Asians, but not in overall individuals and Caucasians. More multicenter studies with larger sample sizes are required for further investigation.

## INTRODUCTION

Breast cancer is a complex and heterogeneous disease and has become a challenging health issue confronted by women worldwide [1, 2]. Previous studies have indicated that obesity is one of the risk factors for postmenopausal women with BC; in addition, weight gain is associated with poor prognosis of premenopausal BC [3, 4]. However, the exact pathogenesis of obesity in the occurrence and development of BC is still unclear. In recent years, leptin, a hormone secreted by adipocytes, has been recognized as one of the plausible mechanisms for carcinogenesis in obese individuals [5].

Leptin is the product of the ob gene that plays a pivotal role in regulating food intake, energy expenditure and neuroendocrine function; furthermore, high levels of leptin have been frequently observed in obese subjects [6, 7, 8]. Several published studies have shown that leptin can regulate endothelial cell proliferation and promote angiogenesis; moreover, it has an association with progression and poor survival of BC [9, 10, 11]. Leptin exerts it physiological action through the leptin receptor which is overexpressed in BC [12]. Genetic variations in some obesity-related genes have been demonstrated to affect BC risk by the levels and functioning of leptin [13, 14, 15]. Over the past few decades, two obesity-related gene polymorphisms have been most commonly reported: LEP G2548A rs7799039, and LEPR Q223R rs1137101. Leptin G2548A polymorphism, located in the promoter region of the leptin gene, has been demonstrated to correlate with variations in serum leptin levels, degree of obesity, as well as cancer susceptibility [16, 17]. LEPR Q223R polymorphism alters amino acid change from neutral to positive that could affect the functionality of the receptor and modifies its signaling capacity [18, 19].

Nevertheless, the studies about rs7799039 and rs1137101 polymorphisms with the risk of BC at present were still controversial: the study by Liu et al. [20] discovered that no significant association between rs7799039 polymorphism and BC risk; while Yan et al. [21] found the Leptin G2548A gene polymorphism played an important role in BC susceptibility, especially among Caucasians. Similarly, results of previous publications for LEPR Q223R rs1137101 and BC risk were also inconsistent [22, 23, 24]. As some new studies published, we conducted this systemic meta-analysis to clarify the role of genetic variants among leptin and leptin receptor in the occurrence and development of BC.

## RESULTS

### Characteristics of included studies

**Figure 1:**
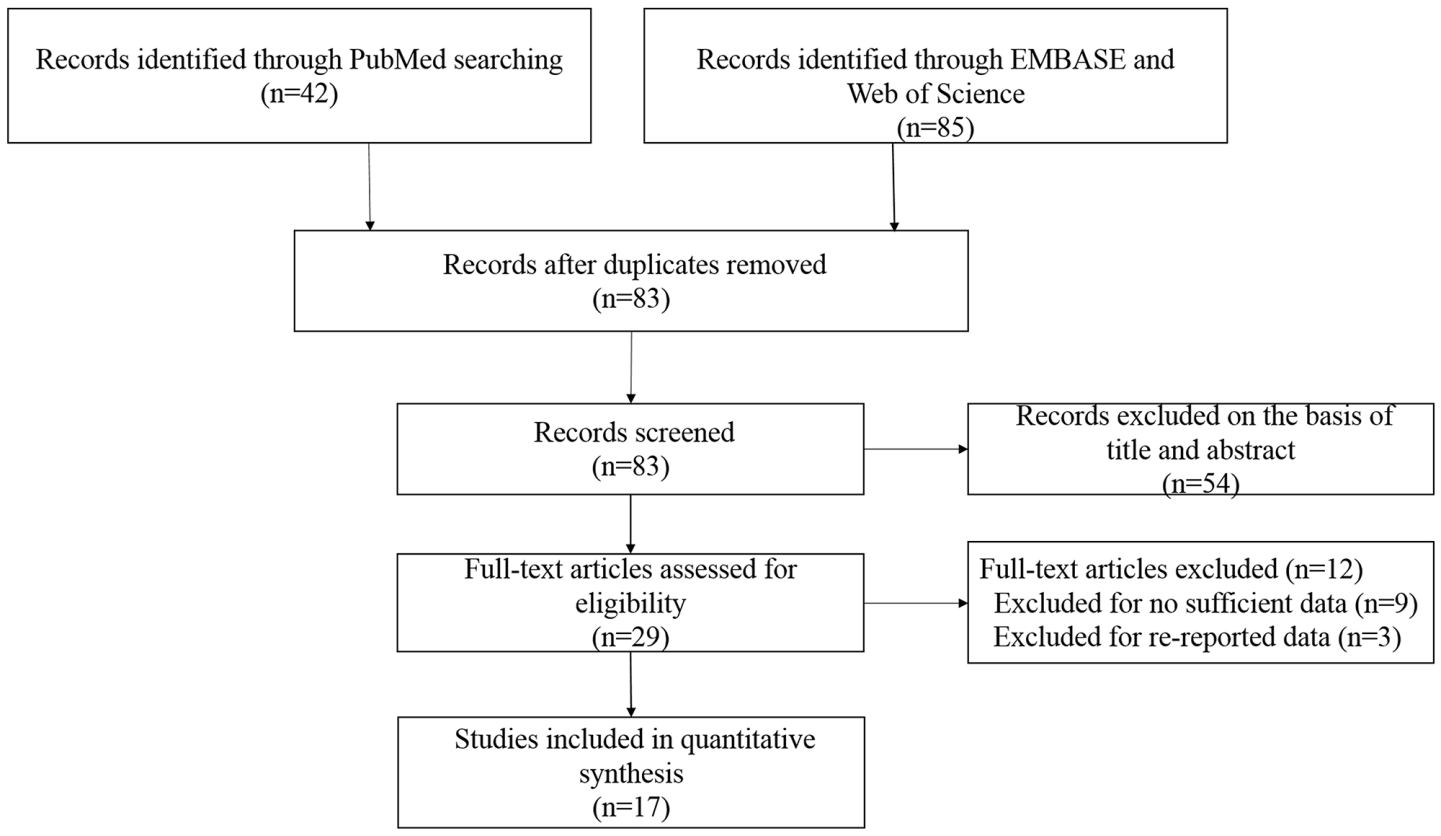
Flow diagram of the selection of the studies in this meta-analysis

The complete search process is presented in Figure 1. According to the search strategy described in the methods and materials section, 127 publications were preliminaries identified. Then we removed duplicate articles, and eighty-three records remained. After reading titles and abstracts of all the studies, we further excluded fifty-four articles that were obviously unrelated. Then, a total of twenty-nine studies were subject to full-text examination. Consequently, eight eligible studies about LEP G2548A polymorphism and thirteen articles about LEPRQ223R polymorphism were included in our meta-analysis. The characteristics of the eligible studies are presented in Table 1. All these included studies were in accordance with the pathological diagnostic criteria of BC and all the papers were published between 2006 and 2017.

**Table 1:** Characteristics of studies included in the meta-analysis

First author	Year	Country	Ethnicity	Genotyping method	Number (case/control)	Sources of controls	HWE (*P* value)
LEPG2548A (rs7799039)							
Rodrigo et al. [32]	2017	Sri Lanka	Caucasian	PCR	80/80	PB	0.890
Cleveland et al. [33]	2010	USA	Caucasian	PCR	1059/1101	PB	0.118
Morris et al. [26]	2013	Mexico	Mixed	PCR	130/189	HB	0.940
Rostami et al. [34]	2015	Iran	Caucasian	PCR-RFLP	203/171	HB	0.383
Mahmoudi et al. [35]	2015	Iran	Caucasian	PCR-RFLP	45/41	PB	0.930
Karakus et al. [36]	2015	Turkey	Caucasian	PCR	199/185	PB	0.407
Snoussi et al. [25]	2006	Tunisia	Mixed	PCR-RFLP	308/222	Unknown	0.063
Mohammadzadeh et al. [37]	2015	Iran	Caucasian	PCR-RFLP	100/100	HB	0.065
LEPRQ223R (rs1137101)							
Snoussi et al. [25]	2006	Tunisia	Mixed	PCR-RFLP	308/222	Unknown	0.162
Woo et al. [38]	2006	Korean	Asian	PCR	45/45	HB	0.513
Gallicchio et al. [39]	2007	USA	Caucasian	Taqman	53/872	PB	0.260
Han et al. [40]	2008	China	Asian	PCR	240/500	HB	0.0009
Okobia et al. [41]	2008	Nigeria	African	PCR-RFLP	209/209	HB	0.704
Teras et al. [27]	2009	USA	Caucasian	SNP stream	648/659	PB	0.090
Cleveland et al. [33]	2010	USA	Caucasian	PCR	1059/1098	PB	0.333
Nyante et al. [42]	2011	USA	Mixed	PCR	1972/1775	PB	0.219
Kim et al. [43]	2012	Korean	Asian	PCR	390/447	HB	0.975
Mahmoudi et al. [35]	2015	Iran	Caucasian	PCR-RFLP	45/41	PB	0.730
Wang et al. [44]	2015	China	Asian	PCR-RFLP	150/128	PB	0.074
Rodrigo et al. [32]	2017	Sri Lanka	Caucasian	PCR-RFLP	80/80	PB	0.000
Mohammadzadeh et al. [45]	2014	Iran	Caucasian	PCR-RFLP	100/100	HB	0.693

HWE: Hardy-Weinberg equilibrium for controls; PCR: polymerase chain reaction; PCR-RFLP: polymerase chain reaction-restriction fragment length polymorphism; PB: population-based study; HB: hospital-based study.

### Meta-analysis results

**Figure 2:**
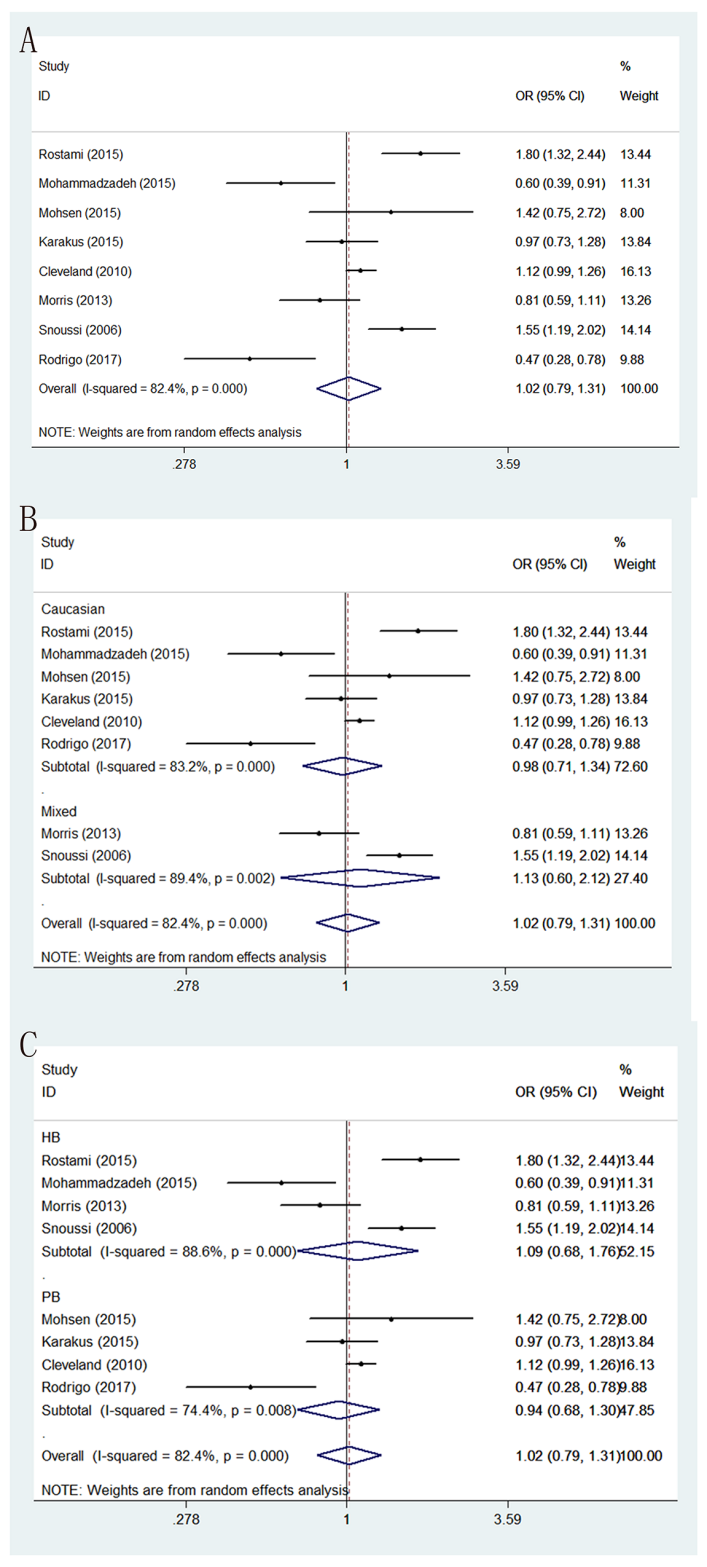
Forest plots of associations between rs7799039 and breast cancer risk in the allele contrast genetic model **(A)** the overall populations; **(B)** stratification by ethnicity; **(C)** stratification by sources of controls.

Genotype, distribution and allele frequency of the two polymorphisms are shown in Table 2, and the main results of this meta-analysis are presented in Table 3. For LEP G2548A polymorphism, eight eligible studies with 2,124 BC patients and 2,089 cancer-free controls were finally identified. In general, we did not observe statistically significant association between LEP G2548A polymorphism and BC risk under the genetic models of AA versus GG, GA versus GG, AA+GA versus GG, AA versus GA+GG and G-allele versus A-allele (OR = 1.105 with 95 % CI 0.699-1.749, OR = 0.991 with 95 % CI 0.857-1.147, OR = 1.081 with 95 % CI 0.942-1.241, OR = 1.074 with 95 % CI 0.692-1.668 and OR = 1.02 with 95 % CI 0.792-1.134, respectively). In subgroup analysis by sources of controls, we also found no significant association between LEP G2548A polymorphism and the risk of BC among these five genetic models. Similarly, further stratified analysis by ethnicity showed no significant correlation between LEP G2548A polymorphism and BC susceptibility in all the ethnic groups (Table 3) (Figure 2).

**Table 2:** Genotype distribution and allele frequency of LEPG2548A (rs7799039) and LEPRQ223R (rs1137101) polymorphisms in cases and controls

First author	Genotype (N)									Allele frequency (N)		
		Case				Control			Case		Control	
	Total	AA	AG	GG	Total	AA	AG	GG	A	G	A	G
LEPG2548A (rs7799039)												
Rodrigo et al.	80	32	43	5	80	53	24	3	107	53	130	30
Cleveland et al.	1059	226	492	341	1101	180	561	360	944	1174	921	1281
Morris et al.	130	22	71	37	189	46	95	48	115	145	187	191
Rostami et al.	203	115	64	24	171	63	77	31	294	112	203	139
Mahmoudi et al.	45	27	11	7	41	17	19	5	65	25	53	29
Karakus et al.	199	49	105	45	197	47	98	40	203	195	192	178
Snoussi et al.	308	37	152	119	222	11	99	112	226	390	121	323
Mohammadzadeh et al.	100	36	55	9	100	52	45	3	127	73	149	51
LEPRQ223R (rs1137101)												
Snoussi et al.	308	98	145	65	222	102	90	30	341	275	294	150
Woo et al.	45	0	12	33	45	0	8	37	12	78	8	82
Gallicchio et al.	53	14	24	15	872	278	443	151	52	54	999	745
Han et al.	240	33	41	166	500	12	78	410	107	373	102	898
Okobia et al.	209	46	107	56	209	56	107	46	199	219	219	199
Teras et al.	648	128	332	181	659	125	314	211	588	694	564	736
Cleveland et al.	1059	173	521	355	1098	187	551	360	867	1231	925	1271
Nyante et al.	1972	494	952	526	1775	416	847	485	1940	2004	1679	1817
Kim et al.	390	8	88	294	447	6	91	350	104	676	103	791
Mahmoudi et al.	45	19	25	1	41	17	18	6	63	27	52	30
Wang et al.	150	20	25	105	128	3	19	106	65	235	25	231
Rodrigo et al.	80	65	9	6	80	60	6	14	139	21	126	34
Mohammadzadeh et al.	100	25	56	19	100	54	40	6	106	94	148	52

**Table 3:** Meta-analysis results

	OR	95%CI	P (OR)	Heterogeneity		Effects model	*P*(Begg)	*P*(Egger)
				I^2^	*P*			
LEPG2548A (rs7799039)								
Total								
A VS G	1.02	0.79-1.31	0.878	82.4%	0.000	R	0.266	0.511
AA VS GG	1.11	0.70-1.75	0.669	71.3%	0.001	R	0.386	0.405
GA VS GG	0.99	0.86-1.15	0.908	12.2%	0.335	F	0.902	0.571
AA+GA VS GG	1.08	0.94-1.24	0.276	46.1%	0.072	F	0.711	0.506
AA VS GA+GG	1.07	0.69-1.67	0.750	84.7%	0.000	R	0.386	0.480
Stratification by ethnicity								
Caucasian								
A VS G	0.98	0.71-1.34	0.888	83.2%	0.000	R	-	-
AA VS GG	1.05	0.64-1.71	0.860	63.7%	0.017	R	-	-
GA VS GG	0.92	0.77-1.08	0.304	0.0%	0.703	F	-	-
AA+GA VS GG	1.02	0.87-1.19	0.817	23.8%	0.255	F	-	-
AA VS GA+GG	1.03	0.62-1.70	0.919	86.1%	0.000	R	-	-
Mixed								
A VS G	1.23	0.60-2.12	0.71	89.4%	0.002	R	-	-
AA VS GG	1.39	0.28-6.90	0.69	90.6%	0.001	R	-	-
GA VS GG	1.27	0.94-1.71	0.11	33.1%	0.221	F	-	-
AA+GA VS GG	1.21	0.65-2.25	0.55	76.0%	0.041	R	-	-
AA VS GA+GG	1.27	0.31-5.11	0.74	89.6%	0.002	R	-	-
Stratification by sources of controls								
Population-based control								
A VS G	0.94	0.68-1.30	0.696	74.4%	0.008	R	-	-
AA VS GG	1.22	0.98-1.52	0.08	22.0%	0.281	F	-	-
GA VS GG	0.92	0.77-1.10	0.34	0.00%	0.711	F	-	-
AA+GA VS GG	1.00	0.85-1.18	0.99	0.00%	0.842	F	-	-
AA VS GA+GG	0.98	0.53-1.81	0.95	84.3%	0.000	R	-	-
Hospital-based control								
A VS G	1.09	0.68-1.76	0.711	88.6%	0.000	R	-	-
AA VS GG	1.13	0.42-3.09	0.806	85%	0.000	R	-	-
GA VS GG	1.18	0.90-1.53	0.229	27.9%	0.245	F	-	-
AA+GA VS GG	1.14	0.69-1.88	0.617	67.4%	0.027	R	-	-
AA VS GA+GG	1.18	0.52-2.67	0.698	88.7%	0.000	R	-	-
LEPRQ223R (rs1137101)								
Total								
G VS A	0.93	0.76-1.23	0.450	87.4%	0.000	R	0.69	0.702
GG VS AA	0.88	0.59-1.30	0.508	85.5%	0.000	R	0.537	0.680
GA VS AA	1.02	0.78-1.34	0.867	75.4%	0.000	R	0.837	0.864
GG VS AA+GA	0.93	0.74-1.15	0.488	75.2%	0.000	R	0.855	0.764
GA+GG VS AA	0.94	0.69-1.28	0.693	84.0%	0.000	R	0.373	0.681
Stratification by ethnicity								
Caucasian								
G VS A	1.09	0.83-1.43	0.534	81.8%	0.000	R	-	-
GG VS AA	1.14	0.67-1.96	0.627	79.0%	0.000	R	-	-
GA VS AA	1.25	0.92-1.69	0.147	53.7%	0.055	R	-	-
GG VS AA+GA	1.05	0.70-1.58	0.801	76.0%	0.001	R	-	-
GA+GG VS AA	1.20	0.84-1.71	0.315	71.7%	0.004	R	-	-
Asian								
G VS A	0.53	0.33-0.86	0.009	80.0%	0.002	R	-	-
GG VS AA	0.23	0.09-0.59	0.002	62.8%	0.068	R	-	-
GA VS AA	0.29	0.12-0.70	0.006	51.3%	0.128	R	-	-
GG VS AA+GA	0.64	0.51-0.79	0.000	49.5%	0.115	F	-	-
GA+GG VS AA	0.24	0.10-0.61	0.003	62.2%	0.071	R	-	-
Stratification by sources of controls								
Population-based control								
G VS A	0.89	0.76-1.05	0.173	72.5%	0.001	R	-	-
GG VS AA	0.84	0.61-1.15	0.267	68.6%	0.004	R	-	-
GA VS AA	0.98	0.87-1.10	0.671	8.50%	0.364	F	-	-
GG VS AA+GA	0.88	0.70-1.11	0.276	68.1%	0.005	R	-	-
GA+GG VS AA	0.94	0.85-1.05	0.301	42.9%	0.105	R	-	-
Hospital-based control								
G VS A	0.93	0.50-1.72	0.82	93.1%	0.000	R	-	-
GG VS AA	0.96	0.21-4.45	0.96	93.3%	0.000	R	-	-
GA VS AA	0.87	0.29-2.63	0.80	90.4%	0.000	R	-	-
GG VS AA+GA	0.96	0.57-1.64	0.89	68.1%	0.005	R	-	-
GA+GG VS AA	0.84	0.23-3.44	0.79	93.7%	0.000	R	-	-

F: fixed effects model; R: random effects model.

**Figure 3:**
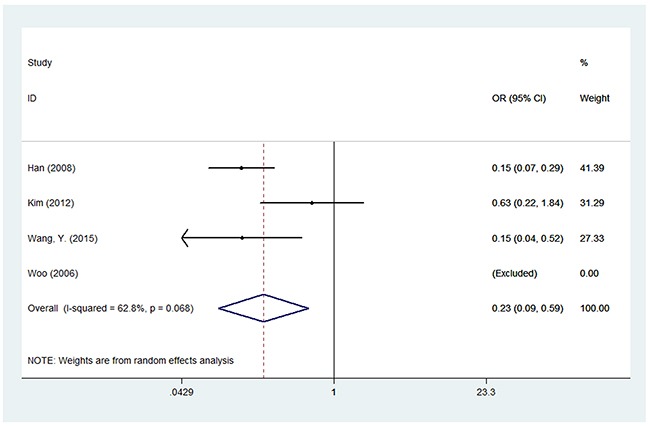
Forest plot of associations between rs1137101 and breast cancer risk among Asians in the homozygote genetic model

For LEPR Q223R polymorphism, thirteen studies with 5,282 cases and 6,140 controls were used to assess the association between this genetic polymorphism and BC susceptibility. We failed to find a significant association between this polymorphism and BC risk among the five genetic models in overall populations. However, in the subgroup analysis by ethnicity, we found LEPR Q223R polymorphism was associated with a decreased risk of BC among Asians under the five genetic models: G versus A (OR = 0.532, 95% CI = 0.311-0.856, *P* = 0.009), GG versus AA (OR = 0.233, 95% CI = 0.092–0.59, *P* = 0.002), GA versus AA (OR = 0.294, 95% CI = 0.124–0.699, *P* = 0.006), GG versus AA+AG (OR = 0.635, 95% CI = 0.521–0.787, *P* < 0.001) and GA+GG versus AA (OR = 0.242, 95% CI = 0.097–0.607, *P* = 0.003), while no meaningful correlation was observed in Caucasians. In addition, we did not observe any association between LEPR Q223R polymorphism and BC risk among the subgroups of population-based controls or hospital-based controls (Table 3) (Figure 3).

### Sensitivity analysis

**Figure 4:**
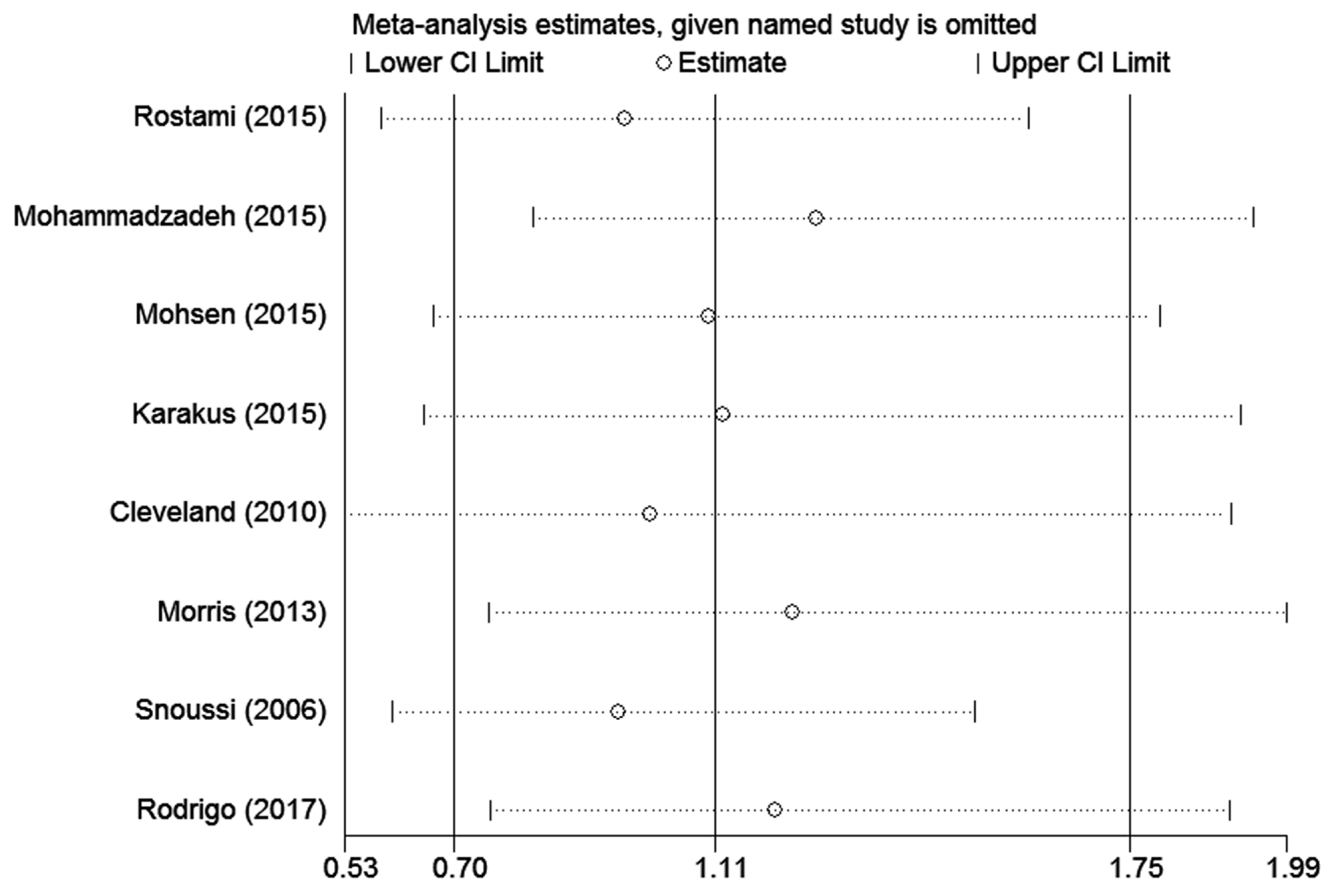
Sensitivity analyses of associations between rs7799039 and breast cancer risk in the homozygote genetic model

**Figure 5:**
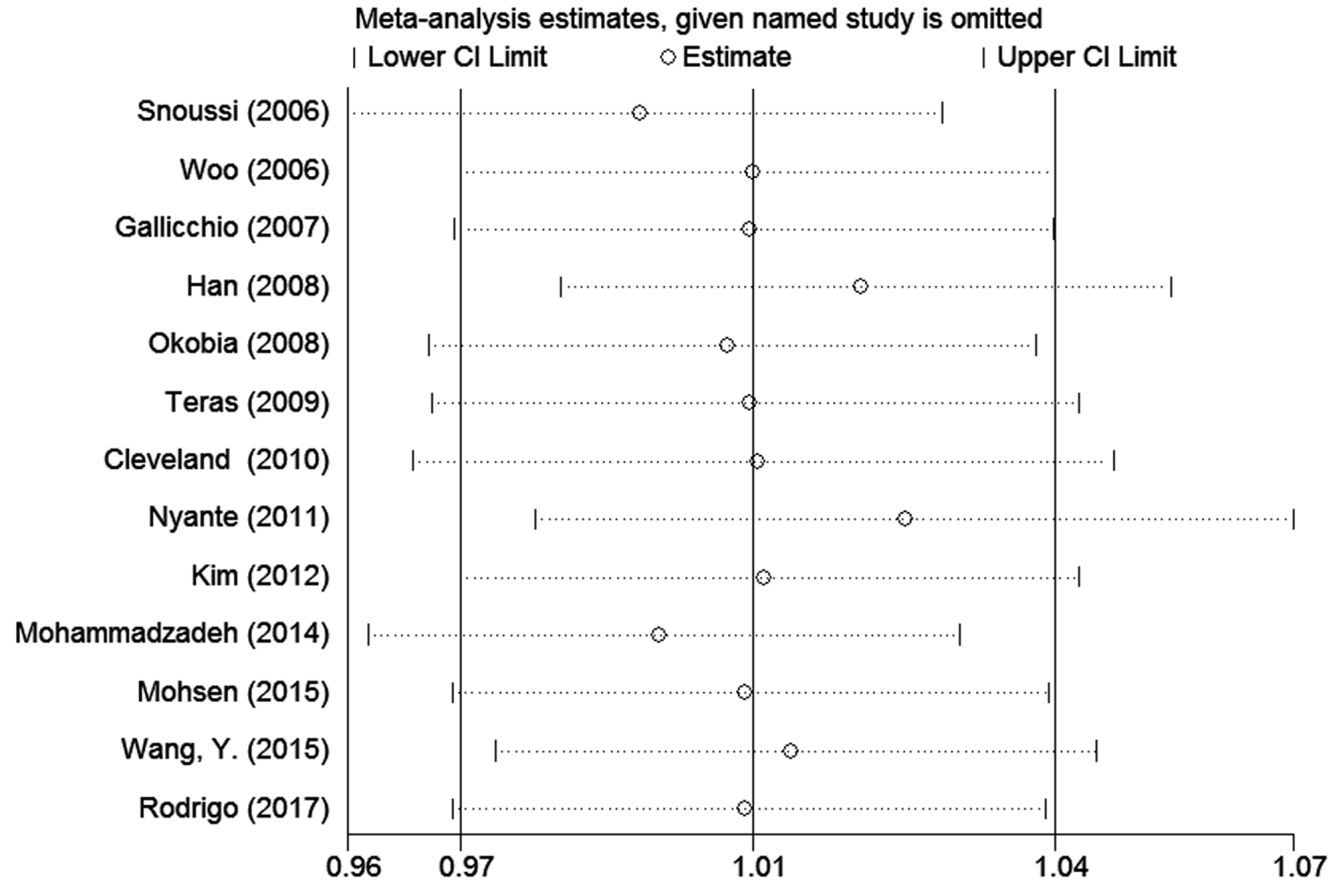
Sensitivity analyses of associations between rs1137101and breast cancer risk in the homozygote genetic model

To assess the stability of the meta-analysis results, sensitivity analysis was performed by deleting one study at a time. After the sensitivity analysis, there was no statistically significant change in the pooled ORs, indicating that our results were reasonable and reliable (Figures 4 and 5).

### Heterogeneity analysis

To determine the heterogeneity among studies in this meta-analysis, we used Q statistic. If the Q test has a *P* value of < 0.1, we consider significant heterogeneity existed and select random-effects model to perform related statistical analysis; if not, we would use fixed-effects model to carry out our research (Table 3).

### Publication bias

Begg's test, Egger's test and funnel plot were carried out to check the publication bias of our studies. All *P* values of Begg's test and Egger's test were greater than 0.05 (*P* > 0.05) (Table 3). Besides, no obvious asymmetry could be found in the funnel plot. Therefore, the results did not suggest any evidence of publication bias in this current meta-analysis.

## DISCUSSION

With the continuous development of society and the progress of technology, especially the improvement in the field of medicine, the knowledge about BC is constantly updating as time goes on. Recently, obesity has been considered as one of the related factors for BC risk [3, 4]. However, the exact pathogenesis of obesity about this malignancy is still the world's unsolved mystery. Previous published studies have demonstrated that leptin, an adipocyte-derived satiety hormone, is associated with the proliferation, angiogenesis, progression, and poor survival in BC cells, especially in higher grade tumors [5, 7, 9]. Two obesity-related gene polymorphisms (rs7799039, and rs1137101) have been recognized as contributors to BC risk through regulating the level and activity of leptin. Leptin G2548A polymorphism in the promoter region of the leptin gene is a potential source of polymorphism affecting gene expression [16, 17]. LEPR Q223R polymorphism occurs in a region which encodes the extracellular domain of the leptin receptor, acts the function of the receptor, and impairs the ability of leptin to bind to its receptor [18, 19]. To date, the association between rs7799039 and rs1137101 polymorphisms and BC susceptibility remains inconclusive. Therefore, in the cause of a more precise evaluation, we performed this meta-analysis.

LEP G2548A polymorphism, located in the 5’ region of the LEP gene, is implicated in transcription [17]. Several studies have shown that the variants might elevate the serum leptin level through transcriptional level [18]. Snoussi et al. [25] showed that polymorphisms in LEP gene are associated with increased BC risk as well as disease progress, while no association of rs7799039 polymorphism with the risk of BC was observed by another study [26]. We observed no association between LEP G2548A polymorphism and BC risk in general populations, and similar results could be seen in the stratified analysis by ethnicity and sources of controls. In the meta-analysis by Liu et al. [20], three studies were included and the result showed that no significant association between rs7799039 polymorphism and BC risk. Nevertheless, Yan et al. [21] found that the rs7799039 polymorphism plays an important role in BC susceptibility, especially in Caucasians; however, there are some mistakes in her study. First, the study of Vairaktaris et al. [18] is about the association of leptin G2548A polymorphism with increased risk of oral cancer, but not BC. Second, the data in the study of Teras et al. [27] did not seem in accordance with the data provided in their original publication. Compared with them, our study includes two new studies, which will expand the sample size and thus get a more precise evaluation.

Leptin exerted its downstream functioning through binding to leptin receptor [19]. For LEPR Q223R polymorphism, our study showed that no association between this polymorphism and BC risk in overall populations. However, in the subgroup analysis by ethnicity, we found LEPR Q223R polymorphism was associated with a decreased risk of BC among Asians, but not in Caucasians. The differences between Asians and other races may be partly due to the different genetic backgrounds and environments or lifestyles [28]. The same result was found in the meta-analysis by Liu et al. [21] in 2011. Wang et al. [22] observed significant associations between LEPR Q223R polymorphism and BC risk limiting the analysis to studies with controls in agreement with HWE in 2015. Compared with previous published meta-analysis, there was thirteen eligible studies in our study, so our result was more reliable with the larger sample size.

Several potential limitations of this meta-analysis should be addressed. First, our study was conducted without adjustment for several potential confounding variables, because of lacking information including age, smoking status, drinking status, environmental factors, and other habits related to lifestyle, which are known to have significant effects on the risk of BC [29, 30]. Second, there was no study based on Asians background between LEP G2548A polymorphism and the risk of BC, so more studies from different ethnic group should be performed to make our conclusions more persuasive. Last but not least, as a multi-factorial disease, the effect of gene–gene and gene–environmental interactions was not estimated in our study [31].

In summary, LEP G2548A polymorphism has no relationship with BC susceptibility, while LEPR Q223R polymorphism could decrease BC risk in Asians, but not in general individuals and Caucasians. However, given the limited sample size, more multicenter studies with larger sample sizes are necessary to clarify the relationships of these polymorphisms with BC risk.

## MATERIALS AND METHODS

### Literature and search strategy

We searched PubMed and EMBASE and Web of Science database up to May 1, 2017. The searching strategy was as follows: (breast cancer OR breast carcinoma) AND (polymorphism OR variant OR genotype OR SNP) AND (leptin OR LEP OR LEPR OR leptin gene receptor). The searching was conducted without the limitations of language. Besides, the references of retrieved studies were further examined to identify additional eligible publications.

### Inclusion criteria

The studies included in the meta-analysis must meet all the following inclusion criteria: (1) clinical trials studying the association between LEP G2548A, or LEPR Q223R polymorphism and the risk of BC; (2) case-control design; (3) sufficient data for calculation of odds ratio (OR) with confidence interval (CI); (4) all the BC subjects in case groups must be pathologically confirmed. Accordingly, the following exclusion criteria were also used: (1) not case-control studies; (2) abstracts, comments, reviews, meta-analyses, and duplicated publications; (3) no detailed genotyping data; (4) the source of cases and controls, and other essential information were not provided; (5) duplicate studies.

### Data extraction

The data from the included studies were independently extracted by two investigators, and discrepancies were resolved through discussion by the research team. The following information was extracted: the first author, year of publication, country of origin, ethnicity, genotyping method, sources of controls, number of cases and controls, and *P* value for Hardy-Weinberg equilibrium (HWE).

### Statistical analysis

Pooled odds ratios (ORs) and 95% confidence intervals (CIs) were used to measure the association between these two polymorphisms and BC risk among five genetic models: an allele contrast genetic model, a homozygote genetic model, a heterozygote genetic model, a dominant genetic model, and a recessive genetic model. Heterogeneity among studies was evaluated by I^2^ test and Q test. For I^2^ test, the criteria for heterogeneity were as follows: I^2^ < 25%, no heterogeneity; 25%-75%, moderate heterogeneity; I^2^ > 75%, high heterogeneity. If the *P* value of the Q test was < 0.1, the random-effects model was used; if not, we would select the fixed-effects model to carry out our research. Sensitivity analysis was performed by deleting one study at a time. To evaluate the publication bias, we used Begg's test, Egger's test and funnel plot. Subgroup analysis was performed according to ethnicity and sources of controls. The *P* value for Hardy-Weinberg equilibrium (HWE) was calculated by the chi-square test in the control group in each study. All statistical analyses were performed using STATA version 10.0 software (Stata Corp LP, College Station, TX, USA). All *P* values were two sided, and *P* < 0.05 was considered statistically significant.
